# Effectiveness of Recovery Colleges: A Nonrandomised Clinical Trial Across Two Years Using Propensity Score Matching: Efficacité des collèges de rétablissement: essai clinique non randomisé sur deux ans utilisant l'appariement par score de propension

**DOI:** 10.1177/07067437261449914

**Published:** 2026-05-14

**Authors:** Marloes M.C. van Wezel, Robin L.A. Smits, Christien Muusse, Ben F.M. Wijnen, Dike van de Mheen, Mike Slade, Claire Henderson, Hans Kroon

**Affiliations:** 1 Tranzo Scientific Centre for Care and Wellbeing, 120694 School of Social and Behavioural Sciences, Tilburg University, Tilburg, the Netherlands; 2 Department of Reintegration and Community Care, Trimbos Institute, Utrecht, the Netherlands; 3 Department of Epidemiology, 26063 Data, Evaluation & Monitoring, Trimbos Institute, Utrecht, the Netherlands; 4 Center of Economic Evaluation & Machine Learning, Trimbos Institute, Utrecht, the Netherlands; 5 School of Health Sciences, 14278 Institute of Mental Health, University of Nottingham, Nottingham, UK; 6 Faculty of Nursing and Health Sciences, Health and Community Participation Division, Nord University, Namsos, Norway; 7 Health Service and Population Research Department, King's College London Institute of Psychiatry, Psychology and Neuroscience, London, UK; 8 4958 South London and Maudsley NHS Foundation Trust, London, UK

**Keywords:** empowerment, mental health, peer support, recovery, severe mental illness

## Abstract

**Background:**

Recovery colleges (RCs) facilitate peer-supported communities where people with lived experience of mental disruption learn collaboratively, aiming to foster empowerment and personal recovery. While existing (qualitative) research relates RC attendance to positive outcomes, high-quality multi-college quantitative studies evaluating RC effectiveness are scarce.

**Aims:**

We examine the extent to which RC attendance impacts empowerment (primary outcome) and other recovery-related outcomes.

**Method:**

In this nonrandomised clinical trial (May 2022 to January 2025), RC partakers from four Dutch RCs were compared with controls over 2 years, in annual data collections. Linear mixed model analyses were conducted to investigate interactions between group and time. Exploratory analyses investigated distinguishing characteristics of RC partakers. This study was embedded in a larger project and was pre-registered (clinicaltrials.gov: #NCT05620212). Academic and experiential researchers collaborated in the design, recruitment, and analysis.

**Results:**

The sample comprised 91 RC partakers and 182 matched controls. RC partakers faced severe or persistent mental health challenges and attended RCs in multiple capacities (e.g., student and visitor/volunteer). Outcomes were mostly stable over time and did not differ between groups. Hence, no group-specific changes over time were found, as shown by interaction terms that were not statistically significant (e.g., empowerment: estimate T1*RC= 0.02, *95% CI=* −0.08–0.13, estimate T2*RC= −0.001, *95% CI=* −0.11–0.11).

**Conclusions:**

Despite promising qualitative evidence of RC effectiveness, our study found no differences between RC partakers and matched controls in quantitative recovery-related outcomes over time. Factors of influence may be possible pre-study effects, unmeasured confounding, or limitations in how standardised questionnaires capture recovery experiences. Methodologically, the findings raise questions about the operationalisation of effectiveness in the context of personal recovery and flexible, co-created practices such as RCs. Importantly, while this study could not establish *measurable* effectiveness, this does not automatically imply that RC attendance cannot *meaningfully* contribute to recovery.

## Plain Language Summary Title

Study evaluating if recovery colleges have effects on recovery by comparing survey scores of people who do vs. do not attend them across two years

**Plain Language Summary:**

**What is the topic of the study?** At recovery colleges, people who experience mental disruption can work on recovery. Partakers share experiences and learn from each other in self-help workshops , social meeting places, and/or as (voluntary) employee.

**Why was the study done?** Recovery colleges aim to support recovery. However, high-quality evaluations are lacking. Therefore this study examines whether attending a recovery college has effects on recovery.

**What did the researchers do?** 273 individuals experiencing mental disruption participated. We compared 91 people who took part in a recovery college (recovery college partakers) with 182 people who did not. For a fair comparison, we matched each recovery college partaker with two participants who did not attend, but were similar on key characteristics, such as age or diagnoses. Participants (both recovery college partakers and people who did not take part in a recovery college) completed surveys about their mental health and recovery at the study start, and one and two years later.

**What did the researchers find?** All participants reported similar empowerment, quality of life, mental health, loneliness, and self-stigma. Scores were similar across groups and also stable over time.

**What do the findings mean?** Based on personal stories, other studies describe benefits of recovery college attendance such as feeling more confident and socially supported. Our survey study did not find effects, which could mean that there is no measurable effect of recovery college attendance. It could also be that effects have already occurred before the study: recovery college partakers already attended the recovery college before the study started. Some suggest that surveys where people score their experiences on a scale do not fully capture what people go through. Importantly, while the study did not find measurable effects, this does not rule out that people can benefit from attending a recovery college. We advise collaboration with lived experience researchers.

## Graphical abstract

**Graphical abstract:**
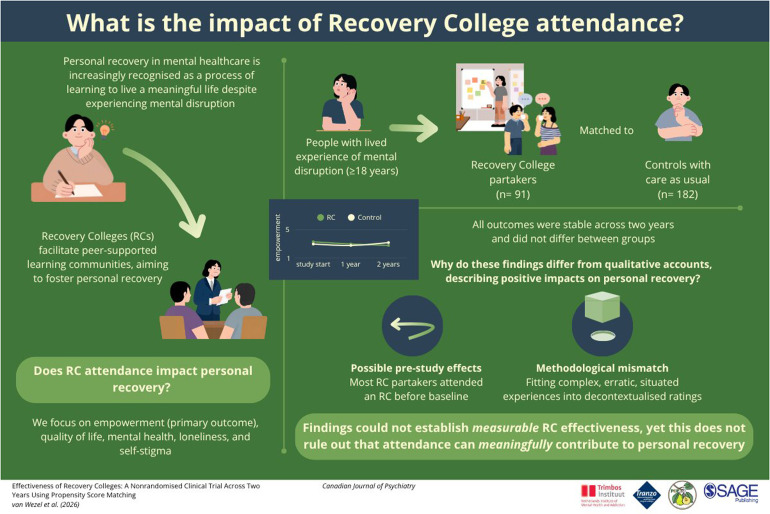
This is a visual representation of the abstract.

## Résumé

**Contexte:**

Les collèges de rétablissement (CR) favorisent la création de communautés soutenues par les pairs, au sein desquelles des personnes ayant vécu des troubles mentaux apprennent de manière collaborative, dans le but de favoriser l’autonomisation et le rétablissement personnel. Bien que les recherches (qualitatives) existantes établissent un lien entre la fréquentation des CR et les résultats positifs, les études quantitatives multicollégiales de grande qualité évaluant l’efficacité des CR sont rares.

**Objectifs:**

Nous examinons dans quelle mesure la fréquentation des CR influe sur l’autonomisation (résultat principal) et d’autres résultats liés au rétablissement.

**Méthode:**

Au cours de cet essai clinique sans répartition aléatoire (de mai 2022 à janvier 2025), les participants aux CR issus de quatre CR néerlandais ont été comparés à un groupe témoin sur une période de deux ans, dans le cadre de collectes de données annuelles. Des analyses en modèle linéaire mixte ont été effectuées pour étudier l’interaction entre le groupe et le temps. Des analyses exploratoires ont examiné les caractéristiques distinctives des participants aux CR. Cette étude s’inscrivait dans un projet plus vaste et a fait l’objet d’un enregistrement préalable (clinicaltrials.gov : #NCT05620212). Les chercheurs universitaires et issus de la pratique ont collaboré à la conception, au recrutement et à l’analyse.

**Résultats:**

L’échantillon comprenait 91 participants aux CR et 182 témoins appariés. Les participants aux CR étaient confrontés à des problèmes de santé mentale graves ou persistants et fréquentaient les CR à divers titres (par exemple, en tant qu’étudiants et visiteurs/bénévoles). Les résultats sont restés globalement stables au fil du temps et ne différaient pas entre les groupes. Par conséquent, aucun changement propre à un groupe n’a été observé au fil du temps, comme le montrent les termes d’interaction qui n’étaient pas statistiquement significatifs (p. ex., l’autonomisation : estimation T1*CR=0,02, *IC à 95%*=-0,08 – 0,13, estimation T2*CR=-0,001, *IC à 95%*=-0,11 – 0,11).

**Conclusions:**

Malgré des preuves qualitatives prometteuses de l’efficacité des CR, notre étude n’a révélé aucune différence entre les participants aux CR et les témoins appariés en ce qui concerne les résultats quantitatifs liés au rétablissement au fil du temps. Les facteurs d’influence peuvent être des effets possibles avant l’étude, des facteurs de confusion non mesurés ou des limites dans la manière dont les questionnaires standardisés rendent compte des expériences de rétablissement. Sur le plan méthodologique, ces résultats soulèvent des questions sur l’opérationnalisation de l’efficacité dans le contexte du rétablissement personnel et des pratiques flexibles et co-créées comme les CR. Il est important de noter que, bien que cette étude n’ait pas pu établir d’efficacité *mesurable*, cela n’implique pas automatiquement que la participation aux CR ne puisse pas contribuer de manière *significative* au rétablissement.

## Introduction

Internationally, recovery is increasingly acknowledged within mental healthcare services as an active learning process emphasising empowerment^1–4^ rather than merely a biomedical process of symptom reduction.^5^ Recovery colleges (RCs) illuminate this transformation in mental healthcare, facilitating peer-supported communities where people with lived experience of mental disruption (i.e., peers) collaboratively learn in recovery-related self-help courses, peer-to-peer interactions and volunteering.^6–8^ RCs are proposed as inspirational examples of recovery-oriented practices^5,9,10^ and are increasingly implemented worldwide.^11^ RC philosophy aligns with an emancipatory movement criticising paternalistic mental healthcare and advocating for centralising lived experiences.^12,13^ As Patricia Deegan, a lived experience pioneer, described:

“Choice, […] role models, […] developing and exercising a voice, opportunities for bettering one's life – these are the features of a human interactive environment that support the transition from not caring to caring, from surviving to becoming an active participant in one's own recovery process”.^14^

### Initial Evidence

Research on RC effectiveness remains scarce.^15–19^ High-quality quantitative studies are limited, and existing research often suffers from methodological limitations.^15,19,20^ To our knowledge, only one pre-post evaluation investigated the effects of RC attendance on empowerment,^21^ though several others examined related constructs such as personal recovery^22,23^ or mental wellbeing,^24–26^ or evaluated online RC offerings.^27,28^ These evaluations and qualitative work^29^ suggest positive impacts on empowerment. Furthermore, research (predominantly qualitative) relates RC attendance to recovery-related outcomes such as experienced support^30,31^ and reduced self-stigma.^23,30,32^ Meta-analyses evaluating courses that many RCs facilitate (e.g., Wellness Recovery Action Planning, WRAP^33^ and Honest, Open, Proud, HOP^34^) also suggest effectiveness. Effects of RC attendance have been suggested to sustain in the long term (e.g., after 1 year^29^).

This study addresses the call for longitudinal, controlled studies acquiring higher quality evidence of RC effectiveness,^15,35^ investigating:

RQ: Does RC attendance impact empowerment (primary outcome), quality of life, mental health, loneliness, and self-stigma?

## Methods

### Design

This nonrandomised controlled trial used propensity score matching to approximate conditions of a randomised controlled trial (RCT).^36^ RC partakers were compared to matched controls from a large, representative Dutch panel of adults (≥ 18 years) facing severe mental illness (SMI), hereafter “the panel.”^37^

### Procedure

Data were collected from May 2022 to January 2025, and also served an economic evaluation (under review^20^). Participants (RC partakers and controls) completed questionnaires online (LimeSurvey) or on paper every 6 months for 2 years, resulting in five assessments. Effectiveness measures for this study were included annually, labelled T0 (baseline), T1 (12 months), and T2 (24 months).

The study builds on a protocol of mixed-methods research on the meaning and effectiveness of RCs^20^ (amendments in Appendix A, Table A1). All procedures comply with the Helsinki Declaration of 1975 (revised 2013) and were approved by the Ethics Review Board of Tilburg University (#TSB_RP390). Participants signed informed consent after being briefed. No data was analysed before data collection ended. Reporting follows elements from STROBE^38^ and CONSORT^39^ guidelines, due to the matched cohort design approximating an RCT.

Academic and experiential researchers (also RC partakers) collaborated throughout the project.^20^ Although the role of experiential researchers was more prominent in qualitative studies, they also contributed to the quantitative design, recruitment and interpretation. For this study, a co-research session was hosted to contextualise findings.

### Participants and Setting

RCs vary internationally, being mostly co-produced with healthcare providers in Anglophone countries vs. mostly peer-run in the Netherlands.^8^ In this study, RC partakers were recruited (on- and offline) at four Dutch RCs (RC1 – RC4). All offered established courses, such as WRAP, and co-created activities. Some RCs facilitated activities at self-managed locations, others in community centres. They also varied in scale (ranging from one to seven locations), organisation (independent foundation, hosted by a mental healthcare or sheltered/supported living organisation) and volunteering opportunities. The four RCs were selected based on expert opinions, were based on similar philosophies, and conformed to the core tasks of Dutch RCs.^8^ RC partaker recruitment was terminated when the required sample size of 125 (for a power of 80%) was comfortably achieved.^20^ The panel (controls) was always open for registration and actively recruited new members on- and offline, through mental healthcare facilities and community services.

For all participants, received care and support could regard outpatient care (e.g., general practitioner, psychiatrist), inpatient care in (psychiatric) hospitals, community care and/or informal support, with varying frequency and intensity. Some participants did not receive care (anymore).

### Group Eligibility

Group eligibility (RC partakers vs. controls) was based on baseline status and not blind. Random allocation was undesirable because it would contradict the open-to-all accessibility of RCs, which emphasises self-chosen attendance. Eligible RC partakers (≥ 18 years) were active at one of the selected RCs at T0 as course students, visitors (of social meeting points), retreat students (taking part in multi-day course with overnight stay), volunteers and/or employees. Eligible controls did explicitly not attend any RC or peer-supported activities at T0 (if they did, they were ineligible for either group and excluded from analysis). Completion of at least the baseline assessment and one follow-up (T1 or T2) was required to be included in data analysis.

### Outcomes

All outcomes were self-reported. Empowerment (primary outcome) was measured with three subscales—confidence and purpose, connectedness and self-management—from the Netherlands Empowerment List (NEL^40^; number of items = 23, *α_T0_=* 0.93, *α_T1_=* 0.94, *α_T2_=* 0.93). Secondary outcomes were quality of life (Maastricht QoL scale^41^; number of items = 6, *α_T0_=* 0.85, *α_T1_=* 0.86, *α_T2_=* 0.85), mental health (MHI-5^42^; number of items = 5, *α_T0_=* 0.87, *α_T1_=* 0.89, *α_T2_=* 0.85), loneliness (DeJong Gierveld Loneliness Scale^43^; number of items = 11, *α_T0_=* 0.92, *α_T1_=* 0.91, *α_T2_=* 0.90) and self-stigma (ISMI-10^44^; number of items = 10, *α_T0_=* 0.76, *α_T1_=* 0.80, *α_T2_=* 0.72). To compute loneliness scores, dichotomised scores were used;^43^ reliability scores were based on the 5-point Likert answers. Internal consistency was acceptable (α ≥ .70) to excellent (α ≥ .90).^45^

### Matching

Propensity score matching using the nearest neighbour method was performed to account for potential confounding^46^ (R package *MatchI*^47^). The propensity score was estimated using logistic regression, and participants were matched in a 1:2 ratio without replacement (matching variables: Appendix B, Table B1). To maintain a parsimonious matching model, diagnostic categories were clustered into seven clusters based on clinical similarity (e.g., depression and bipolar disorder grouped as mood disorders) and sample size considerations (e.g., combining disorders with low and comparable frequencies across groups into an “other” category).

### Analyses

After matching, linear mixed model analyses were conducted, using R packages *lme4*^48^ and *lmerTest.*^49^ Group, timepoint and their interaction term were added as fixed effects, participant ID as a random effect. If interactions were not statistically significant, a model including main effects of group and timepoint was run. Estimated marginal means (EMMs) were computed to provide model-based averages for each group at each timepoint. These estimates account for the repeated measurements within participants, ensuring fair between-group comparisons across timepoints. Data was analysed based on intention-to-treat. Between-group differences in descriptives were tested using independent samples t-tests or Mann–Whitney U-tests (non-normal data distributions) for continuous variables, and Chi-squared or Fisher's exact tests (if any cell <5 observations) for categorical variables. *P*-values were corrected for multiple testing using the Holm–Bonferroni procedure,^50^ with an adjusted *P*-value < 0.05 indicating statistical significance. Additionally, effect sizes were computed using Cohen's d^51^ to provide information about the magnitude and practical relevance of potential effects, with values of 0.30, 0.50 and 0.80 typically interpreted as small, moderate and large effects, respectively.

### Sensitivity and Exploratory Analyses

Mixed models are generally robust to missing data.^52^ However, we conducted an imputation-based sensitivity analysis to determine whether conclusions remained consistent regardless of the missing-data approach, using multiple imputation (R package *mice*^53^). Additionally, we examined potential confounding by (mental) healthcare use, considering dichotomous mental healthcare use (yes or no) and total healthcare costs at T0. Healthcare costs (in Euros) were indexed to 2022 (the study's starting year); the reference period was 3 months.

Finally, we explored whether RC partakers had different characteristics than those who did not attend an RC, comparing RC partakers with *all* (including non-matched) eligible controls, using statistical testing of descriptives.

## Results

**Figure 1.**
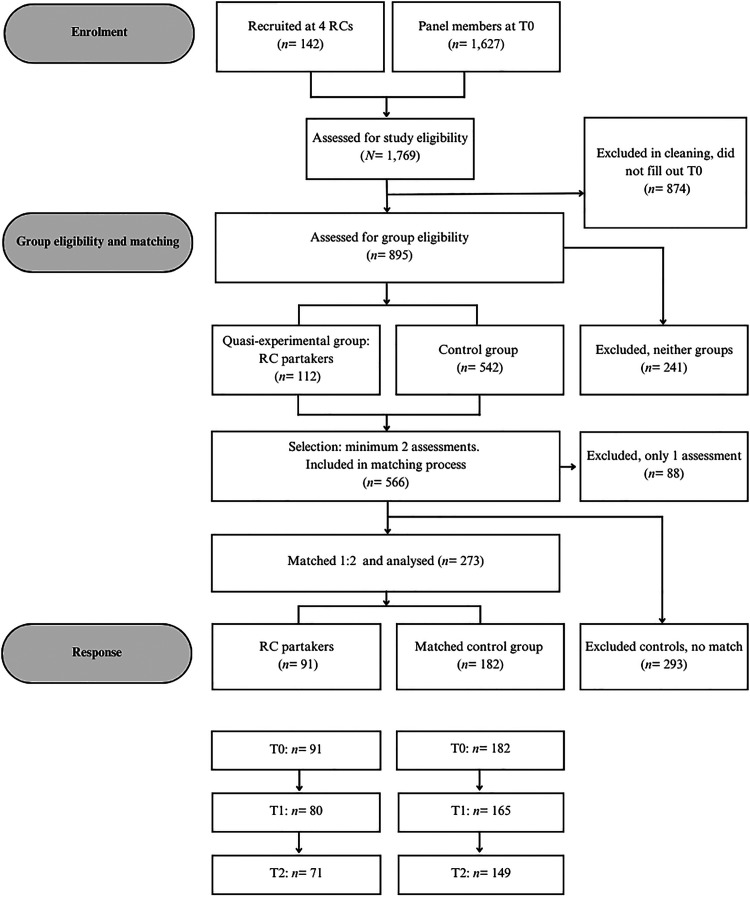
CONSORT flowchart.

In total, 566 out of 1,769 participants (existing panel members and newly recruited participants) were eligible for inclusion (Figure 1). All 91 RC partakers were matched to 182 eligible controls (38.3% of total 475 eligible controls), composing the definite sample (*N=* 273).

### Descriptives

At the study start, participants were 48 years old on average (*SD=* 12.05) and 75.0% identified as female. Eleven participants reported a gender identity different from their sex. Participants had 2–3 diagnoses of mental disorders on average (*M=* 2.51, *SD=* 1.49, *Range=* 0–7), and 71.4% had more than one. Almost all participants (97.4%) experienced mental health challenges for more than 2 years, 73.3% used psychotropic medication, 9.6% had been admitted in the past 12 months to a psychiatric hospital, and 67.0% used mental healthcare (most prevalent: outpatient mental healthcare clinic [28.2%], Flexible Assertive Community Treatment [18.3%] and private practitioners [16.9%]). About one in four was employed (working 23.84 h per week, *SD=* 10.66), and 46.5% volunteered (5.99 h per week, *SD=* 4.27). Post-matching, standardised mean differences (SMDs) between groups (RC – control) were acceptable for all matching variables (<0.10^36^) except for healthcare costs (*SMD=* 0.14; descriptives of matching variables in Table 1). Descriptives other than the matching variables are specified per group in Table 2.

**Table 1.** Matching Variable Descriptives Pre- and Post-Matching, Including Standardised Mean Differences (SMD) between RC and Control Group.

		Pre-Matching		Post-Matching	
	RC *n=* 91	Control *n=* 475	Total *N=* 566	Control *n=* 182	Total *N=* 273
**Sex**	**n (%)**	**n (%)**	**SMD**	**n (%)**	**SMD**
Female	69 (76)	316 (67)	0.22	144 (79)	−0.08
Male	21 (23)	159 (34)	−0.25	38 (21)	0.05
Else	1 (1)	0 (0)	0.11	0 (0)	0.11
**Age**					
Mean (SD)	47.84 (11.28)	54.03 (11.85)	−0.55	47.83 (12.46)	0.00
Range	23–73	23–83	−	23–83	−
Unknown	0	5	−	1	−
**Origin**					
Dutch	69 (78)	400 (86)	−0.20	141 (80)	−0.04
Non-Dutch	19 (22)	65 (14)	0.18	35 (20)	0.04
Unknown	3 (-)	10 (-)	0.07	6 (-)	0.00
**Education**					
Primary or secondary	13 (17)	103 (24)	−0.21	25 (16)	0.02
Vocational	29 (38)	168 (39)	−0.08	57 (36)	0.01
Academic	34 (44)	153 (35)	0.11	72 (45)	−0.05
Else	1 (1)	11 (3)	0.15^a^	5 (3)	0.03^a^
Unknown	14 (-)	40 (-)	−	23 (-)	−
**Diagnoses**					
Neurocognitive developmental disorders	29 (32)	103 (22)	0.22	57 (31)	0.01
Psychotic disorders	13 (14)	128 (27)	−0.36	25 (14)	0.02
Mood disorders	54 (59)	279 (59)	0.01	113 (62)	−0.06
Anxiety disorders	33 (36)	163 (34)	0.04	65 (36)	0.01
Trauma and stress	39 (43)	131 (28)	0.31	74 (41)	0.04
Personality disorders	35 (39)	114 (24)	0.30	67 (37)	0.03
Other disorders	29 (32)	82 (17)	0.31	53 (29)	0.06
**Healthcare costs (€)**					
Mean (SD)	2,287 (3,821)	1,760 (3,957)	0.14	1,771 (3,280)	0.14
Range	0–27,261	0–55,937	−	0–28,985	−

a For SMDs of education, levels else + unknown were combined. RC = Recovery college.

**Table 2.** Baseline Descriptives.

	Total *n=* 273	RC *n=* 91	Control *n=* 182
**Gender**	**n (%)**	**n (%)**	**n (%)**
Female	132 (75)	56 (73)	76 (77)
Male	35 (20)	18 (23)	18 (18)
Non-binary	3 (2)	1 (1)	2 (2)
Else	3 (2)	1 (1)	2 (2)
Rather not say	2 (1)	1 (1)	1 (1)
Unknown (NA)	97 (-)	14 (-)	83 (-)
**Number of diagnoses**			
Mean	2.51	2.55	2.49
SD	1.49	1.55	1.47
Range	0–7	0–7	0–6
#N more than 1	195 (71)	67 (74)	128 (70)
**Duration of mental health problems**			
<1 year	7 (3)	1 (1)	6 (3)
1–2 years	0 (0)	0 (0)	0 (0)
>2 years	260 (97)	87 (99)	173 (97)
Unknown (NA)	6 (-)	3 (-)	3 (-)
**Use of psychotropic medication**			
Yes	200 (73)	59 (65)	141 (77)
No	73 (27)	32 (35)	41 (23)
**Mental healthcare use**			
Yes	183 (67)	69 (76)	114 (63)
No	90 (33)	22 (24)	68 (37)
**Type of mental healthcare use**			
Hospitalisation in past 12 months	26 (10)	12 (13)	14 (8)
Outpatient mental healthcare clinic	77 (28)	33 (36)	44 (24)
Sheltered or supported housing	38 (14)	17 (19)	21 (11)
FACT	50 (18)	12 (13)	38 (21)
Primary care	26 (10)	9 (10)	17 (9)
Private practitioners	46 (17)	20 (22)	26 (14)
**Paid employment**			
Yes	68 (25)	18 (20)	50 (27)
No	205 (75)	73 (80)	132 (73)
**Employment hours (*n=* 68)**			
Mean	23.84	22.33	24.38
SD	10.66	10.66	10.72
Range	4–47	4–40	4–47
**Volunteering**			
Yes	127 (47)	53 (58)	74 (41)
No	146 (53)	38 (42)	108 (59)
**Volunteering hours (*n=* 127)**			
Mean	6.00	6.19	5.85
SD	4.27	4.60	4.05
Range	0–20	0–20	1–16

*Note.* NA = not available; FACT = flexible assertive community treatment; RC = Recovery college.

#### Dropouts

On average, participants completed 2.70 (*SD=* 0.46) assessments, with 70.3% completing all three (i.e., completers). A Mann–Whitney U-test showed that assessment completion did not differ between RC partakers and controls (*M_RC_=* 2.66, *M_control_=* 2.73, *W=* 7735, *P=* 0.26). In terms of the matching variables, non-completers reported more “unknown” or “other” education levels (non-completers: 44.4% vs. completers: 3.7%) and less vocational (non-completers: 19.8% vs. completers: 36.5%) or academic (non-completers: 25.9% vs. completers: 44.3%) education levels (*χ*2 (3)*=* 71.54, *adjusted P<*0.001). Missing data per outcome are specified for each timepoint in Table 3.

**Table 3.** Estimated Marginal Means (EMM) and Missingness of Primary and Secondary Outcomes by Group at the Three Assessments.

	RC (*n=* 91)			Control (*n=* 182)		
Outcome	EMM	95% CI	Missing (%)	EMM	95% CI	Missing (%)
**Primary**						
*Empowerment* ^a^	3.48	3.35–3.60		3.34	3.25–3.43	
Baseline (T0)	3.45	3.32–3.58	0	3.32	3.23–3.41	1
One-year (T1)	3.52	3.38–3.65	12	3.37	3.27–3.46	11
Two-year (T2)	3.46	3.33–3.60	23	3.34	3.24–3.43	19
**Secondary**						
*Quality of life* ^b^	4.55	4.33–4.77		4.53	4.38–4.69	
Baseline (T0)	4.41	4.18–4.65	0	4.50	4.33–4.66	1
One-year (T1)	4.62	4.38–4.86	12	4.49	4.32–4.66	11
Two-year (T2)	4.64	4.39–4.88	23	4.61	4.43–4.78	18
*Mental health* ^c^	53.78	50.18–57.38		51.13	48.58–53.67	
Baseline (T0)	52.35	48.42–56.29	0	50.17	47.38–52.95	1
One-year (T1)	55.47	51.43–59.51	12	51.80	48.95–54.66	12
Two-year (T2)	53.51	49.36–57.67	23	51.40	48.49–54.31	19
*Loneliness* ^d^	6.55	5.87–7.22		6.54	6.07–7.02	
Baseline (T0)	6.61	5.88–7.35	1	6.41	5.89–6.93	1
One-year (T1)	6.45	5.70–7.20	12	6.44	5.91–6.97	12
Two-year (T2)	6.53	5.75–7.30	23	6.80	6.26–7.35	20
*Self-stigma* ^e^	2.08	2.00–2.17		2.21	2.15–2.27	
Baseline (T0)	2.07	1.98–2.16	0	2.21	2.14–2.27	1
One-year (T1)	2.08	1.99–2.17	12	2.21	2.15–2.28	13
Two-year (T2)	2.10	2.01–2.20	25	2.21	2.14–2.27	20

a Scores range from 1 to 5, with higher scores indicating better empowerment.

b Scores range from 1 to 7, with higher scores indicating better quality of life.

c Scores range from 0 to 100, with higher scores indicating better mental health.

d Scores range from 0 to 11, with higher scores indicating more loneliness.

e Scores range from 1 to 4, with higher scores indicating more self-stigma.

*Note*. Overall EMMs are based on the LMM with main effects of group and time, EMMs per assessment are based on the LMM with interaction terms included. Model estimates from the LMM with main effects are provided in Appendix D.

RC = Recovery college; LMM = linear mixed model.

#### Recovery College Attendance

At T0, most RC partakers (62.6%) attended RC1 (vs. RC2: 9.9%, RC3: 14.3%, RC4: 15.4%; two participants attended two RCs). RC partakers were involved as course students (76.9%), visitors (38.5%), volunteers (37.4%), retreat students (23.1%) and/or employees (11.0%). More than half were active in multiple capacities, most frequently as both course student and visitor or volunteer. On average, RC partakers attended the RC 1.78 (*SD=* 1.16, *Range=* 1–5) times per week. RC volunteers worked 6.77 h per week (*SD=* 7.83, *Range=* 0–44), RC employees worked 16.00 h (*SD=* 11.3, *Range=* 1–32) per week. Of the 55 RC partakers who reported the start year of their RC attendance (lower response due to a technical error), more than half (55.9%) had been active at the RC for 0–2 years (of which 6.6% just started), 25.4% had been active for 3–5 years and 11.9% had been active for 6+ years. Some controls also attended an RC during the study (Appendix C, Table C1).

### Primary Outcome: Empowerment

Empowerment remained stable for both RC partakers and controls (e.g., RC group: *EMM_T0_=* 3.45, *EMM_T1_=* 3.52, *EMM_T2_=* 3.46; Table 3) and both groups reported comparable levels of empowerment (e.g., on T1, between-group difference in EMMs was 0.15 on a 5-point scale). Hence, there were no group-specific changes over time, as indicated by group-by-time interactions that were not statistically significant (estimate T1*RC= 0.02, *95% CI=* −0.08–0.13, estimate T2*RC= −0.001, *95% CI=* −0.11–0.11; Table 4).

**Table 4.** Results of Linear Mixed Models (Primary and Secondary Outcomes) with Group-by-Time Interaction Effects.

Outcome	Estimate	SE	CI 95%	*P*	Adjusted P	Cohen's D
**Primary**						
*Empowerment*						
Intercept	3.32	0.05	3.23–3.41	−	−	-
T1*RC	0.02	0.06	−0.08–0.13	0.66	1.00	0.08
T2*RC	−0.001	0.06	−0.11–0.11	0.99	1.00	−0.003
**Secondary**						
*Quality of life*						
Intercept	4.50	0.08	4.33–4.66	−	−	-
T1*RC	0.22	0.10	0.02–0.41	0.03	0.29	0.42
T2*RC	0.12	0.10	−0.09–0.32	0.26	1.00	0.22
*Mental health*						
Intercept	50.17	1.42	47.39–52.94	−	−	-
T1*RC	1.48	1.89	−2.22–5.18	0.43	1.00	0.15
T2*RC	−0.07	1.97	−3.94–3.80	0.97	1.00	−0.01
*Loneliness*						
Intercept	6.41	0.26	5.90–6.93	−	−	-
T1*RC	−0.19	0.35	−0.88–0.50	0.59	1.00	−0.10
T2*RC	−0.48	0.37	−1.20–0.24	0.19	1.00	−0.26
*Self-stigma*						
Intercept	2.21	0.03	2.14–2.27	−	−	-
T1*RC	0.01	0.05	−0.08–0.09	0.90	1.00	0.02
T2*RC	0.03	0.05	−0.06–0.13	0.48	1.00	0.14

*Note.* Baseline (T0) and control are references. Statistical significance: adjusted *P*<0.05. Cohen's d: 0.20 (small), 0.50 (moderate), 0.80 (large).

RC = Recovery college.

### Secondary Outcomes

Temporal trends in secondary outcomes also remained stable for both groups (except for a minor, but statistically significant, increase in quality of life at T2 for both groups: Appendix D, Table D1). All group-by-time interaction effects were not statistically significant, indicating that trends over time were similar for both RC partakers and controls (e.g., Quality of life: estimate T1*RC= 0.22, *95% CI=* 0.02–0.41, estimate T2*RC= 0.12, *95% CI=* −0.09–0.32; Table 4).

### Sensitivity Analyses

All sensitivity analyses on imputed data, controlled for baseline dichotomous mental healthcare use (67.0% users vs. 33.0% non-users, comparable ratios in RC and control group) and controlled for total healthcare costs led to similar conclusions (e.g., empowerment in imputed dataset: estimate T1*RC= 0.02*, 95% CI=* −0.08–0.13, estimate T2*RC= 0.01*, 95% CI=* −0.10–0.11; *all adjusted P's≥* 0.29; Appendix E, Tables E1–3).

### Exploratory Analysis: Characteristics of Recovery College Partakers

The exploratory comparison of RC partakers (*n=* 91) with all eligible pre-matching controls (*n=* 475), highlighted some differences (Appendix F, Table F1). RC partakers were significantly younger (*M_RC_=* 47.84, *M_control_=* 54.03, *M_dif_=* −6.19, 95% CI= −8.77 – −3.62, *adjusted P<* 0.001), more often reported a diagnosis in the “other” category (RC: 31.9% vs. control: 17.3%, *adjusted P=* 0.04) and more often volunteered (RC: 58.2% vs. control: 38.7%, *adjusted P=* 0.02) than pre-matching controls. Of the 53 RC partakers that volunteered, 33 (62.3%) did so at the RC.

## Discussion

This is one of the first longitudinal, controlled evaluations of RC effectiveness. Findings showed that temporal trends in self-reported recovery-related outcomes were mostly stable and did not differ for RC partakers and controls. Over the 24-month period, neither group showed significant improvement in empowerment or any of the secondary outcomes (except for a minor significant increase in quality of life, in both groups). While the absence of group-specific changes and improvements overall could suggest a lack of measurable effectiveness, these results should be interpreted in the light of some methodological considerations. For instance, between-group differences may have been attenuated because most RC partakers had already been active in an RC years before baseline, or because both groups received considerable care and support.^54^

One longitudinal, matched-controlled evaluation of recovery education centres (REC) offers a partially different perspective. That study reported improvements in empowerment (and mastery) after enrolment, but only among REC participants (vs. controls) who attended at least 14 h during the 12-month study period.^55^ Yet, interpreting this finding is not straightforward, as it may reflect (a) a novelty effect,^56^ (b) confounding by unmatched variables, such as care use (which was not documented) or level of education (which was higher in the 14+ hours REC group), or (c) a chance finding (due to an increased probability of false positive findings if multiple comparisons are not corrected for^50,57^). In line with our findings, Durbin et al.^55^ found no significant differences between REC partakers and controls on other outcomes.

Although pre- to post-studies^21–23^ and qualitative inquiries^29,35^ (including a triangulated qualitative study we conducted in this same setting^58^) report positive within-person changes in recovery-related outcomes among RC partakers, the broader evidence base suggests that measurable, quantified effects of recovery-oriented interventions are generally small. Both meta-analyses investigating within-person changes in personal recovery (*d=* 0.34)^59^ or between-group effectiveness of peer support (*g=* 0.15)^60^ report small effect sizes that require substantial statistical power to be detected. Furthermore, at a conceptual level, the recovery processes that RCs aim to foster may be challenging to capture through standardised between-group comparisons alone (see Methodological Implications). Taken together, while this quantitative evaluation may not have been able to establish an added *measurable* effect of RC attendance compared to usual care, this does not rule out that RCs can *meaningfully* contribute to personal recovery processes.

### Practical Implications

Although this study provides no conclusive answers on quantitative RC effectiveness, it offers detailed insights into who attend RCs and in what capacity. Our study's participants faced long-standing and complex mental health challenges, reporting a long duration of experienced mental health problems, comorbidity and substantial care use. RC partakers attended an RC for extended periods and in various roles. Furthermore, RC partakers more often volunteered than comparable individuals facing SMI, especially within the RC, underscoring the potential of RCs as places fostering activation and participation.

Also, RC partakers were younger and more often classified in the “other” diagnostic category, corroborating that some groups may be underrepresented in RCs.^61,62^ This raises questions about who RCs reach (depending on context), and how the aspired inclusive culture^7,8^ can be obtained.

### Methodological Implications

Our findings prompt critical reflection on how well questionnaires capture experienced recovery. Several scholars suggest that standardised, pre-defined questionnaires might not sufficiently allow nuanced, accurate reports of experiences, for example, because experiences fall between items or are context dependent.^63–65^ This especially holds for recovery processes, which are non-linear, relational and emergent. Moderate, stable scores as found in our study may therefore reflect averaging, compensatory response strategies of participants to fit complex, erratic experiences into decontextualised ratings,^65^ rather than stable recovery or lack of progress. That said, effects on empowerment for people with SMI have been reported, using the same measurement of empowerment as we used (NEL^66^). As indicated, quantified, measurable effects of RC attendance are likely to be small, and possibly, revealing them requires even larger, more robust designs. In any way, researchers should critically reflect on the extent to which standardised questionnaire responses alone offer accurate insights into lived realities.

### Strengths and Limitations

This study stands out for its longitudinal, propensity score-matched design. RCs were evaluated as integral initiatives encompassing various ways of attendance. Panel data access facilitated a large sample of potential controls, enabling precise matching. Despite randomisation being unfeasible within RCs, this procedure resulted in a high-quality evaluation. Furthermore, embedding the study within a larger research project^20^ offered access to rich data (e.g., mental healthcare use) and integration of experiential knowledge through the co-created, mixed-methods designs and collaboration with a well-established group of experiential researchers.

These strengths also hold limitations. Approaching the RC integrally meant encountering diverse RC partaking: attendance ranged from 1 to 5 times weekly with many RC partakers combining roles. Activity impact likely varies by context and individual factors^58^ (see also in consumer-run organisation context^67^) and besides that, impacts are expected in various, person-specific domains. This heterogeneity and expected small effect sizes required a substantial sample, but dropouts reduced power and may have introduced selection bias regarding education level. Nonetheless, prior RC studies and experiential researchers’ practice-based familiarity with the RC population supported the sample's representativeness, and attrition rates were relatively small and comparable across groups. Finally, the number of newly engaged RC partakers was smaller than anticipated, and rapid developments of Dutch RCs during the study period may have contributed to controls attending RCs too. The number of known contaminations was small (12 of 182), suggesting that any bias introduced is probably limited. Still, because RC attendance was unknown for part of the control group, some additional contamination cannot be ruled out. Future studies may therefore benefit from larger samples and more strict inclusion criteria to ensure clearer between-group differentiation.

### Future Directions

“Effectiveness” in personal recovery, especially within co-created, flexible contexts such as RCs, should be reconsidered. Group-based comparisons may fall short in evaluating how RC attendance can contribute to erratic, meaning-driven recovery processes. Moreover, randomisation contradicts the open-to-all philosophy of RCs. Evaluations of RC effectiveness should accommodate both the individuality of recovery processes and the richness of RC practices.^8,68^ One promising direction involves conducting large-scale, mixed-methods studies (e.g., diary studies) integrating quantitative and qualitative data, offering deeper understanding of how participants interpret and give meaning to their experiences over time.^64^ Given that recovery processes are unique and personal, future research may explore whether incorporating personalised outcome measures (such as goal attainment^69^) yields additional nuance beyond what standardised measures can capture.

Research should also establish a true baseline by including RC partakers when they start attending, though this is challenging (initial RC attendance can feel overwhelming, limiting research participation). By co-designing studies that are engaging, accessible and low-threshold, together with experiential researchers, studies can better align with participants’ realities. In any way, evaluations should prioritise co-creative methods that integrate experiential knowledge, ensuring that lived experiences shape the design and interpretation of findings. We experienced that this approach fosters richer understanding, more awareness of power dynamics and meaning of evaluations for RC partakers: gains that literature also highlights.^17,70^

Finally, retrospective analysis of our existing sample could explore heterogeneity. Pairwise comparisons may uncover meaningful change in RC partakers (compared to their matched controls) obscured by aggregated statistics. While avoiding overgeneralising personal recovery processes, exploratory comparisons could yield insights into factors associated with positive outcomes.

## Conclusion

Despite the growing body of qualitative evidence suggesting RC effectiveness, our longitudinal, controlled quantitative evaluation did not corroborate this. Empowerment and other recovery-related outcomes were mostly stable over time and did not differ for RC partakers and matched controls. While these findings do not provide measurable support for RC effectiveness, methodological and conceptual considerations caution against drawing definite conclusions. At the same time, detailed insights into who attend RCs (individuals facing severe or persistent mental health challenges) and attendance capacities (in varied roles, mostly for longer periods of time) were acquired. The findings raise questions about how effectiveness can be operationalised in the context of personal recovery and flexible, co-created practices such as RCs, and how evaluative approaches can be refined. Importantly, while this study could not establish a *measurable* added effect of RC attendance beyond care as usual, this does not rule out that RC attendance can *meaningfully* contribute to recovery processes.

## Appendix

**Appendix A – Protocol Amendments**

This study was pre-registered in a protocol.^20^ In this appendix, we describe which amendments were made to the protocol and why.

**Table A1.** Rationales for Amendments to the Protocol.

	Protocol	Amendment	Rationale
1	Include satisfaction with care and support as outcome variables	Exclude satisfaction variables and not report them	Care use and informal support varied over time and were not applicable to all participants. Moreover, the satisfaction items lacked a defined time frame in its formulation, unlike the care use items, impeding the identification of valid satisfaction responses from actual care users.
2	Use gender as matching variable	Use biological sex as matching variable	Instead of gender, biological sex was used as one of the matching variables, as few participants (*N=* 11, 4.0%) identified differently from their biological sex, and adequate matching for these participants would be difficult.
3	Use nationality as matching variable	Use origin as matching variable	We used origin instead of nationality as a matching variable, since origin reflects social and cultural identity, whereas nationality is a legal status.
4	Match 1:1	Match 1:2	The availability of the large-scale panel allowed us to compare 1:1 vs. 1:2 matching and select the one with lowest standardised mean differences. Besides, enlarging the control group increases the precision of the estimates in this group and in turn a more precise estimate of group differences.
5	Use single imputation nested in bootstrapping	Use multiple imputation	Single imputation nested in bootstrapping was applied in the economic evaluation that was part of the research project, however we deviated from this approach in the effectiveness study. Integrating single imputation nested in bootstrapping with linear mixed models (LMMs) is computationally intensive and methodologically complex. Therefore, multiple imputation was adopted as a robust alternative.
6	Fit the models without intercept	Fit models with intercept	Although the study lacks randomisation and a true baseline, we included an intercept in the model to enable direct comparison between groups at each time point. Overall group means were subsequently estimated using estimated marginal means.
7	Sensitivity analyses: cross-overs and nature of RC attendance	Not conducted	Due to the sample size of the RC group, these sensitivity analyses were not feasible.

RC = Recovery college.

## Appendix B – Matching Variables

**Table B1.** Description of Included Matching Variables.

Matching Variable	Includes
1. Sex	Sex (female, male, else)
2. Age	Age in years at T0
3. Origin	Origin (Dutch, non-Dutch, unknown)
4. Level of education	Level of education, with four options: primary and secondary (incl. little to no education, primary school, (lower) pre-vocational secondary education), vocational (incl. senior general secondary education, pre-university education, secondary vocational education), academic (university of applied sciences, university) and else or unknown.
5–11. Diagnoses (seven clusters)	
*5. Neurocognitive and developmental disorders*	Autism, Attention-Deficit/Hyperactivity Disorder
*6. Psychotic disorders*	Psychosis, schizophrenia
*7. Mood disorders*	Bipolar mood disorder, depressive mood disorder
*8. Anxiety disorders*	Anxiety, obsessive compulsive disorder
*9. Trauma and stress*	Post-traumatic stress disorder, dissociative identity disorder
*10. Personality disorders*	Personality disorders
*11. Other disorders*	Alcohol addiction, substances addiction, behavioural addiction, eating disorders
12. Healthcare costs (€)	Costs of received outpatient care (e.g., general practitioner, psychiatrist), inpatient care in (psychiatric) hospitals, community care, and psychotropic medication. In Euros, indexed to 2022 (study start), the reference period was 3 months.

## Appendix C – Cross-Over Information

Of the RC partakers, 30 were active at one of the selected RCs at all three assessments, others stopped attending or their attendance fluctuated (details on RC attendance among the RC group in Table C1). Among controls, 12 engaged with an RC during the study after T0 (included RC, *n=* 6, other RC, *n=* 6) and 12 controls had attended an RC in the 12 months before (but not at) T0 (included RC, *n=* 7, other RC, *n=* 5, data on RC attendance was incomplete for 23 controls at T1 and 42 controls at T2).

**Table C1.** RC Attendance of RC Partakers (N= 91) During the Study.

Status	T	N		
		Yes	No	Unknown
Attended at time of assessment				
- Included RC	T0	91	0	0
	T1	51	25	15
	T2	40	27	24
- Other RC	T0	1^a^	90	0
	T1	2	74	15
	T2	2	65	24
Attended in past 12 months but not at time of assessment				
- Included RC	T0	0	91	0
	T1	17	59	15
	T2	7	60	24
- Other RC	T0	0	91	0
	T1	3	73	15
	T2	1	66	24

a Also attended RC1. RC = Recovery college.

## Appendix D – Results Main Effect Model

**Table D1.** Results of Linear Mixed Models with Main Effects of Group and Time.

Outcome	Estimate	SE	CI 95%	*P*	Adjusted P	Cohen's D
**Primary**						
*Empowerment*						
Intercept	3.32	0.05	3.23–3.41	−	−	−
Group	0.13	0.08	−0.01–0.28	0.08	0.86	0.46
T1	0.05	0.03	0.00–0.10	0.04	0.52	0.18
T2	0.02	0.03	−0.04–0.07	0.58	1.00	0.05
**Secondary**						
*Quality of life*						
Intercept	4.46	0.08	4.30–4.62	−	−	−
Group	0.02	0.14	−0.24–0.29	0.87	1.00	0.04
T1	0.06	0.05	−0.03–0.16	0.18	1.00	0.12
T2*	0.15	0.05	0.05–0.24	0.003	0.04*	0.28
*Mental health*						
Intercept	50.01	1.38	47.31–52.71	−	−	−
Group	2.65	2.24	−1.74–7.04	0.24	1.00	0.27
T1	2.13	0.89	039–3.87	0.02	0.22	0.21
T2	1.22	0.92	−0.59–3.03	0.19	1.00	0.12
*Loneliness*						
Intercept	6.48	0.26	5.98–6.98	−	−	−
Group	0.00	0.42	−0.82–0.82	1.00	1.00	0.00
T1	−0.04	0.17	−0.36–0.29	0.82	1.00	−0.02
T2	0.23	0.17	−0.10–0.57	0.18	1.00	0.13
*Self-stigma*						
Intercept	2.20	0.03	2.14–2.27	−	−	−
Group	−0.13	0.05	−0.23 – −0.03	0.01	0.20	−0.53
T1	0.01	0.02	−0.03–0.05	0.72	1.00	0.03
T2	0.01	0.02	−0.03–0.05	0.65	1.00	0.04

*Statistically significant (*adjusted P<*0*.*05). Cohen's d: 0.20 (small), 0.50 (moderate), 0.80 (large).

## Appendix E – Results Sensitivity Analyses

**Table E1.** Results of Linear Mixed Models (Primary and Secondary Outcomes) with Group-by-Time Interaction Effects on Imputed Data.

	Original			Imputed Data					
Outcome	Estimate	SE	CI 95%	Estimate	SE	CI 95%	*P*	Adjusted *P*	Cohen's d
**Primary outcome**									
Empowerment									
Intercept	3.32	0.05	3.23–3.41	3.32	0.05	3.23–3.41	−	−	−
T1*RC	0.02	0.06	−0.08–0.13	0.02	0.05	−0.08–0.13	0.65	1.00	0.08
T2*RC	−0.001	0.06	−0.11–0.11	0.01	0.05	−0.10–0.11	0.91	1.00	0.02
**Secondary outcomes**									
Quality of life									
Intercept	4.50	0.08	4.33–4.66	4.49	0.08	4.33–4.66	−	−	−
T1*RC	0.22	0.10	0.02–0.41	0.21	0.10	0.02–0.40	0.03	0.34	0.39
T2*RC	0.12	0.10	−0.09–0.32	0.11	0.10	−0.08–0.30	0.25	1.00	0.21
Mental health									
Intercept	50.17	1.42	47.39–52.94	50.18	1.41	47.40–52.95	−	−	−
T1*RC	1.48	1.89	−2.22–5.18	1.31	1.84	−2.31–4.92	0.48	1.00	0.13
T2*RC	−0.07	1.97	−3.94–3.80	0.28	1.84	−3.33–3.89	0.88	1.00	0.03
Loneliness									
Intercept	6.41	0.26	5.90–6.93	6.42	0.26	5.91–6.94	−	−	−
T1*RC	−0.19	0.35	−0.88–0.50	−0.17	0.35	−0.85–0.51	0.62	1.00	−0.09
T2*RC	−0.48	0.37	−1.20–0.24	−0.48	0.35	−1.16–0.20	0.17	1.00	−0.25
Self-stigma									
Intercept	2.21	0.03	2.14–2.27	2.21	0.03	2.15–2.28	−	−	−
T1*RC	0.01	0.05	−0.08–0.09	0.01	0.04	−0.08–0.09	0.86	1.00	0.03
T2*RC	0.03	0.05	−0.06–0.13	0.03	0.04	−0.06–0.11	0.51	1.00	0.12

*Note.* Baseline (T0) and control are references. RC = Recovery college.

**Table E2.** Results of Linear Mixed Models (Primary and Secondary Outcomes) with Group-by-Time Interaction Effects Controlled for Mental Healthcare Use (MHC Use, Yes or No, at T0).

	Original			Controlled for MHC Use					
Outcome	Estimate	SE	CI 95%	Estimate	SE	CI 95%	*P*	Adjusted *P*	Cohen's d
**Primary outcome**									
Empowerment									
Intercept	3.32	0.05	3.23–3.41	3.35	0.05	3.24–3.45	−	−	−
T1*RC	0.02	0.06	−0.08–0.13	0.01	0.06	−0.10–0.13	0.79	1.00	0.05
T2*RC	−0.001	0.06	−0.11–0.11	−0.01	0.06	−0.13–0.10	0.80	1.00	−0.05
**Secondary outcomes**									
Quality of life									
Intercept	4.50	0.08	4.33–4.66	4.51	0.09	4.33–4.69	−	−	−
T1*RC	0.22	0.10	0.02–0.41	0.19	0.10	−0.01–0.39	0.06	0.59	0.37
T2*RC	0.12	0.10	−0.09–0.32	0.09	0.11	−0.12–0.30	0.40	1.00	0.17
Mental health									
Intercept	50.17	1.42	47.39–52.94	51.63	1.62	48.47–54.80	−	−	−
T1*RC	1.48	1.89	−2.22–5.18	1.48	1.92	−2.28–5.24	0.44	1.00	0.15
T2*RC	−0.07	1.97	−3.94–3.80	−0.21	2.01	−4.16–3.73	0.92	1.00	−0.02
Loneliness									
Intercept	6.41	0.26	5.90–6.93	6.27	0.30	5.68–6.86	−	−	−
T1*RC	−0.19	0.35	−0.88–0.50	−0.10	0.36	−0.80–0.60	0.78	1.00	−0.05
T2*RC	−0.48	0.37	−1.20–0.24	−0.32	0.37	−1.06–0.41	0.39	1.00	−0.18
Self-stigma									
Intercept	2.21	0.03	2.14–2.27	2.16	0.04	2.09–2.23	−	−	−
T1*RC	0.01	0.05	−0.08–0.09	0.02	0.05	−0.07–0.11	0.60	1.00	0.10
T2*RC	0.03	0.05	−0.06–0.13	0.05	0.05	−0.04–0.14	0.31	1.00	0.21

*Note.* Baseline (T0) and control are references. MHC = Mental healthcare; RC = Recovery college.

**Table E3.** Results of Linear Mixed Models (Primary and Secondary Outcomes) with Group-by-Time Interaction Effects Controlled for Healthcare Use Costs (at T0*)*.

	Original			Controlled for Healthcare Use Costs					
Outcome	Estimate	SE	CI 95%	Estimate	SE	CI 95%	*P*	Adjusted *P*	Cohen's d
**Primary outcome**									
Empowerment									
Intercept	3.32	0.05	3.23–3.41	3.36	0.05	3.26–3.45	−	−	−
T1*RC	0.02	0.06	−0.08–0.13	0.02	0.06	−0.08–0.13	0.66	1.00	0.08
T2*RC	−0.001	0.06	−0.11–0.11	−0.001	0.06	−0.11–0.11	0.99	1.00	0.00
**Secondary outcomes**									
Quality of life									
Intercept	4.50	0.08	4.33–4.66	4.54	0.09	4.36–4.72	−	−	−
T1*RC	0.22	0.10	0.02–0.41	0.22	0.10	0.02–0.41	0.03	0.29	0.42
T2*RC	0.12	0.10	−0.09–0.32	0.12	0.10	−0.09–0.32	0.26	1.00	0.22
Mental health									
Intercept	50.17	1.42	47.39–52.94	51.59	1.50	48.64–54.54	−	−	−
T1*RC	1.48	1.89	−2.22–5.18	1.47	1.89	−2.23–5.17	0.44	1.00	0.15
T2*RC	−0.07	1.97	−3.94–3.80	−0.07	1.97	−3.94–3.80	0.97	1.00	−0.01
Loneliness									
Intercept	6.41	0.26	5.90–6.93	6.31	0.28	5.76–6.87	−	−	−
T1*RC	−0.19	0.35	−0.88–0.50	−0.19	0.35	−0.88–0.50	0.59	1.00	−0.10
T2*RC	−0.48	0.37	−1.20–0.24	−0.48	0.37	−1.20–0.24	0.19	1.00	−0.26
Self-stigma									
Intercept	2.21	0.03	2.14–2.27	2.17	0.03	2.10–2.24	−	−	−
T1*RC	0.01	0.05	−0.08–0.09	0.01	0.05	−0.08–0.09	0.90	1.00	0.02
T2*RC	0.03	0.05	−0.06–0.13	0.03	0.05	−0.06–0.13	0.47	1.00	0.14

*Note.* Baseline (T0) and control are references. RC= Recovery college.

## Appendix F – Results Exploratory Analysis

**Table F1.** Descriptives of RC Partakers vs. Pre-Matching Controls (N= 566).

	RC Partakers (*n=* 91)	Pre-Matching Controls (*n=* 475)	Test	Test Statistic	ΔM (95% CI)	*P*	Adjusted *P*
	n (%)	n (%)		t or χ^2^			
*Matching variables*							
**Sex**			Fisher exact	**-**	**-**	0.02	0.24
Female	69 (76)	316 (66)					
Male	21 (23)	159 (34)					
Else	1 (1)	0					
**Age (*n=* 561)****			Independent samples T-test	−4.76	−6.19 (−8.77 – −3.62)	< 0.001	<0.001
Mean (SD)	47.84 (11.28)	54.03 (11.85)					
Range	23–73	23–83					
Unknown	0	5					
**Origin**			Chi-squared	2.76	-	0.10	
Dutch	69 (78)	400 (86)					
Non-Dutch	19 (22)	65 (14)					
Unknown (NA)	3 (-)	10 (-)					
**Education** ^a^			Chi-squared	5.08	-	0.17	1.00
Primary or secondary	13 (17)	103 (24)					
Vocational	29 (38)	168 (39)					
Academic	34 (44)	153 (35)					
Else	1 (1)	11 (3)					
Unknown (NA)	14 (-)	40 (-)					

	RC Partakers (*n=* 91)	Pre-Matching Controls (*n=* 475)	Test	Test Statistic			
	n (%)	n (%)		t or χ^2^	ΔM (95% CI)	*P*	Adjusted *P*
**Diagnoses**							
Neurocognitive developmental disorders	29 (32)	103 (22)	Chi-squared	3.88	-	0.05	0.54
Psychotic disorders	13 (14)	128 (27)	Chi-squared	5.89	-	0.02	0.21
Mood disorders	54 (59)	279 (59)	Chi-squared	0.00	-	1.00	1.00
Anxiety disorders	33 (36)	163 (34)	Chi-squared	0.06	-	0.81	1.00
Trauma and stress	39 (43)	131 (28)	Chi-squared	7.77	-	0.005	.10
Personality disorders	35 (39)	114 (24)	Chi-squared	7.51	-	0.006	.10
Other disorders*	29 (32)	82 (17)	Chi-squared	9.43	-	0.002	.04
**Healthcare costs (€)** ^b^			Independent samples T-test	1.20	527.07 (−342.96–1,397.09)	0.23	1.00
Mean (SD)	2,287 (3,821)	1,760 (3,957)					
Range	0–27,261	0–55,937					
*OTHER DESCRIPTIVES*							
**Gender**			Fisher exact	-	-	0.16	1.00
Female	56 (73)	166 (60)					
Male	18 (23)	99 (36)					
Non-binary	1 (1)	3 (1)					
Else	1 (1)	9 (3)					
Rather not say	1 (1)	2 (1)					
Unknown (NA)	14 (-)	196					
**Number of diagnoses** ^c^			Independent samples T-test	2.54	0.44 (0.10–0.79)	0.01	0.18
Mean	2.55	2.11					
SD	1.55	1.39					
Range	0–7	0–6					
#N More than 1	67 (74)	289 (61)					

	RC Partakers (*n=* 91)	Pre-Matching Controls (*n=* 475)	Test	Test Statistic	ΔM (95% CI)	*P*	Adjusted *P*
	n (%)	n (%)		t or χ^2^			
**Duration of mental health problems**			Fisher exact	-	-	0.61	1.00
< 1 year	1 (1)	16 (3)					
1–2 years	0 (0)	3 (1)					
> 2 years	87 (99)	441 (96)					
Unknown (NA)	3 (-)	15					
**Use of psychotropic medication**			Chi-squared	8.25	-	.004	.08
Yes	59 (65)	376 (79)					
No	32 (35)	98 (21)					
Unknown (NA)	0 (-)	1 (-)					
**Mental healthcare use**			Chi-squared	7.31	-	.007	.11
Yes	69 (76)	286 (60)					
No	22 (24)	189 (40)					
**Hospitalisation in past 12 months**			Chi-squared	4.67	-	.03	.37
Yes	12 (13)	29 (6)					
No	79 (87)	445 (94)					
Unknown (NA)	0 (-)	1 (-)					
**Paid employment**			Chi-squared	0.00	-	1.00	1.00
Yes	18 (20)	93 (20)					
No	73 (80)	378 (80)					
Unknown (NA)	0 (-)	4 (-)					
**Employment hours** **(*n=* 111)**			Independent samples T-test	−1.55	−4.28 (−9.98–1.42)	0.13	1.00
Mean	22.33	26.61					
SD	10.66	11.14					
Range	4–40	2–64					

	RC Partakers (*n=* 91)	Pre-Matching Controls (*n=* 475)	Test	Test Statistic	ΔM (95% CI)	*P*	Adjusted *P*
	n (%)	n (%)		t or χ^2^			
**Volunteering***			Chi-squared	11.15	-	<0.001	.02
Yes	53 (58)	184 (39)					
No	38 (42)	291 (61)					
**Volunteering hours** **(*n=* 237)**			Independent samples T-test	−1.09	−0.90 (−2.53–0.73)	0.28	1.00
Mean	6.19	7.09					
SD	4.60	7.20					
Range	0–20	0–60					
